# "Knowing" and "Looking"

**Published:** 1902-10-18

**Authors:** 


### The Hospital.

A Journal of

The Medical Sciences and Hospital Administration.

Vol. XXXIII.—No. 838.] Edited by Sir Henry Burdett, K.C.B., and Solomon C. Smith, M.D., M.R.C.P. [October 18, 1902.

### "Knowing" and "Looking."

We referred last week to an introductory lecture in which attention had been called, in a very striking way, to the position of the medical profession regarded as a whole, and in relation to the public and to other callings with which it might be compared; and we may turn to-day to another, which was full of suggestive matter concerning the individual practitioner, and the actual performance of his duties in the sick-room. Dr. Whipham, at St. George's, after dealing with certain matters of detail, of interest chiefly to the school in which they had occurred, took as his text what he rightly described as an aphorism worthy to rank with the aphorisms of Hippocrates, the saying of the late Sir William Gull that "in medicine we make more mistakes by not looking than by not knowing." A saying of this kind, in order to be of any validity, must, of course, like a proverb, be the wisdom of many embodied in the wit of one; and its importance will largely depend upon the extent to which it crystallises, in a form appropriate for mental reference and application, something which is universally perceived to be true, but is not on that account any less liable to be neglected or forgotten. Sir Thomas Watson was not less emphatic than Gull in impressing upon his pupils the importance of testing by direct examination the accuracy of the statements made to them; and in this he was but following the example of many illustrious predecessors. The necessity for such warnings has been to some extent attributable to the admiration often felt by the unskilled for the power of rapid diagnosis which some highly accomplished physicians and surgeons have attained; men who have been said by their admirers to "see at a glance" the nature of the malady from which their patient was suffering, and to prescribe, by a sort of intuition, the appropriate remedy. Such power, in so far as it has really existed, has been the ultimate result of long and laborious study of facts and symptoms, and is not to be acquired in any other manner. The constitutionally inaccurate, or the comparatively inexperienced, if they aim at the accomplishment of similar feats, will only succeed in showing how easily it is possible to jump to inaccurate conclusions. The Apostolic maxim to prove all things, and to hold fast what is good, applies to diagnosis as much as to any other of the tasks of life; and the best physician or the best surgeon will be he who takes nothing for granted, but brings his first impressions to the test of careful and rigorous examination. In precise proportion as he does this will the power of combining rapidity with correctness be added to him.

It is not difficult, in looking round the world, to observe a very definite division of mankind into two classes, with regard to the systematic verification of their mental impressions; some being always painstaking and accurate, others always hasty and superficial; and it is perhaps worth while to inquire to what extent these differences are either innate, or due to conditions which better methods of education might modify or remove. It is at least certain that merely literary education, of the kind which is intended or supposed to impart scholarship, has no specially favourable influence in this direction, of which there can be no better proof than the constant inaccuracy of quotations, an inaccuracy which frequently destroys the appropriateness of the passage quoted, and not uncommonly entirely alters its meaning. It is related that the venerable head of a college in one of the Universities, at the close of a life prolonged beyond the ordinary years of man, summoned the Fellows to his death-bed, and, after wishing them farewell, said, in feeble accents, "Always verify your references." The truism enunciated by Solomon, that in a multitude of counsellers there is safety, has in common parlance been altered into the nonsense that there is *wisdom*, and there is at least one edition of the poems of Charles Wolfe in which the survivors of Sir John Moore are made to gaze on "the face *of the* dead." In the current literature of the day, in the abounding rhodomontade of the pulpit and the platform, and in the almost inconceivable ignorance of many of the smatterers of the press, we are surrounded by powerful influences which make for inaccuracy and vagueness, and which involve no small risk that decisions upon questions important to the public welfare may be based upon errors in respect of fact, and upon fallacies in respect of reasoning. But, to go back to Sir William Gull, the only foundation for knowing is in looking; and the most hopeful form of education is that which will teach the young to look, and to look for themselves instead of trusting to the eyes of others. For this purpose, the best school-book is the book of Nature, and the best form of teaching is that which rests largely upon nature study. Not the least of the evils arising from the existing aggregation of mankind into huge urban communities is the extent to which the book of Nature is thereby closed to the young; and if Bacon were writing to-day he would be likely to add to his enumeration of *idola* those arising from the life of towns, or rather from the exclusion of a wider life which it entails. Solomon sent his sluggard to the ant; and the haste and presumptuousness of the half-educated of our own day could assuredly find no better monitor.

